# Editorial: Immunometabolism and nutritional regulation of intestinal mucosal immunity

**DOI:** 10.3389/fimmu.2024.1365690

**Published:** 2024-01-17

**Authors:** Yingying Feng, Wai San Cheang, Renyou Gan, Xin Wu

**Affiliations:** ^1^ Tianjin Institute of Industrial Biotechnology, Chinese Academy of Sciences, Tianjin, China; ^2^ State Key Laboratory of Quality Research in Chinese Medicine, Institute of Chinese Medical Sciences, University of Macau, Macau, Macau SAR, China; ^3^ Singapore Institute of Food and Biotechnology Innovation (SIFBI), Agency for Science, Technology and Research (A*STAR), Singapore, Singapore

**Keywords:** immunometabolism, nutritional regulation, intestinal mucosal immunity, Th22 cell, IRF8, gut microbes

The intestinal tract is the body’s largest immune organ and front line of defense. Intestinal mucosal injury can lead to intestinal immune dysfunction and immune cell metabolic disorders. Taking nutrients from the daily diet is an effective way to improve intestinal immunity. The present Research Topic was designed to exhibit reviews or original research articles highlighting recent advances in the potential effects of special nutrients and immunometabolism on intestinal mucosal immunity.

Immune cells assume a pivotal role in preserving the functionality of the intestinal mucosa. Among numerous immune cells, T helper cells type 22 (Th22) cells, a subtype of CD^4+^ T cells that can secrete interleukin-22 (IL-22), have been observed at sites of infection and in various autoimmune diseases and have attracted considerable attention. The review of Chen and Yao elucidated the impact on the integrity of the intestinal mucosal barrier by presenting an overview of the molecular structure characteristics and functional effects of Th22 cells and IL-22, as well as explored the targeted treatment approaches and potential therapeutic strategies focusing on the Th22 and IL-22 pathways. As mentioned by the authors, IL-22 could increase the level of mucin secretion and the production of antimicrobial peptides, affect tight junctions and epithelial proliferation, and influence the composition and structure of the gut microbiota to maintain the mucosal barrier. However, clinical samples from patients with active ulcerative colitis (UC) and Crohn’s disease (CD) have demonstrated increased levels of IL-22, indicating the pro-inflammatory properties of IL-22. The review contributes to a deeper understanding of the intricate functionality of Th22 cells and IL-22 that has led to conflicting claims regarding their effects on mucosal barriers. Therefore, further investigation is required to understand the precise role of Th22 cells and IL-22.

In addition to interleukins, immune cells can release various regulators of innate immune receptor signaling to resist pathogen invasion by regulating cell growth and differentiation. Zong et al. paid their attention to the inhibitory effects and potential action mechanisms of interferon regulatory factor 8 (IRF8), a key regulator for interferon (IFN) mediating the clearance of virus-infected cells, in intestinal tract infections by establishing an intestinal damage model caused by porcine epidemic diarrhea virus (PEDV) in piglets. They detected IRF8 expression in the intestine of piglets and found that PEDV infection could activate IRF8 gene expression. By deletion and overexpression of IRF8 and detection of viral copy, cell activity, and inflammatory factor expression, they verified that IRF8 activation could resist PEDV infection via apoptosis and oxidative stress pathways. In this study, Zong et al. provides valuable insights into the IRF8 in PEDV replication and it lays a foundation for developing therapeutic strategies for PEDV-associated diseases.

Gut microbes play a vital role in initiating immunological activation due to their abundance when compared to human cells. Besides, their byproducts that act as signaling molecules can trigger the immune system in the gut mucosa. The review of Fu et al. offers comprehensive insights into the interaction between gut microbial metabolites and the intestinal immune system. As mentioned by the authors, short-chain fatty acids (SCFA) and secondary bile acids can release inflammatory-mediated signals by binding to specific receptors, and tryptophan-derived metabolites can bind to aromatic hydrocarbon receptors (AHR) located on the intestinal mucosa, enhancing the intestinal epithelial barrier. Although numerous studies highlighted the impact of gut microbial metabolites on host immunity, the precise mechanism of this relationship remains elusive. Overall, this review provides potential for more in-depth investigations to unearth mechanisms underlying gut microbiota-host interactions.

Nutrition levels can profoundly affect the development and function of intestine, especially the mucosal immune system and intestinal microbiota. The review of Andres et al. highlighted the importance of understanding the mechanistic effects of undernutrition on the intestinal ecosystem to explore how nutrition and undernutrition impact pediatric intestinal health. The physiology of the intestine in the undernourished state with a disrupted barrier and imbalances in the microbial community, impacts nutrient digestion and absorption in malnourished children. Therefore, the authors emphasized the importance of enteral nutrition in the intestinal development of early infants. As stated, adequate nutrition is intimately linked to intestinal immune function, since it is the key to maintain the function of macrophages, dendritic cells (DCs), and gut associated lymphoid tissue (GALT), secretion of secretory immunoglobulin A (SIgA) and the symbiotic relationships among intestinal epithelial cells (IECs), barrier function, and the associated microbiome. However, at this stage, how specific nutrients or dietary components, such as vitamin D, substrates for the aryl hydrocarbon receptor, protein, carbohydrates, or even food-derived extracellular vesicles, affect intestinal immune system in the malnourished intestine remains unclear. These insights further reinforce the importance of understanding the mechanistic effects of undernutrition on the intestinal ecosystem to better treat and improve long-term outcomes for survivors.

In alignment with this perspective, Cai et al. focused on the role and mechanism of dietary yeast glycoprotein (YG) supplementation on the growth performance, intestinal health and disease resistance of largemouth bass (*Micropterus salmoides*) fed with low-fishmeal diets. Their results showed that dietary YG supplementation to low fishmeal diet enhanced intestinal physical barriers by upregulating the intestinal tight junction protein related genes including claudin1, occludin and zona occludens 2 (ZO-2) and improving the structural integrity of the gut, which may be partially associated with AMP-activated protein kinase (AMPK) signaling pathway. In addition, dietary YG could increase the intestinal anti-inflammatory factors and downregulate pro-inflammatory factors, which may be partially related to the nuclear factor erythroid 2–related factor 2 (Nrf2)/Kelch-like ECH-associated protein 1 (Keap1) signaling pathways. This research is a good example that nutrients can alter barrier function in the malnourished intestine, providing a new strategy for the replacement of fishmeal by plant proteins in aquaculture.

Overall, this Research Topic points towards advanced research on the immunometabolism of intestinal mucosal immunity with its nutritional regulation to achieve a beneficial impact on intestinal mucosal injury.

## Author contributions

YF: Validation, Writing – original draft, Writing – review & editing. WC: Validation, Writing – review & editing. RG: Validation, Writing – review & editing. XW: Validation, Writing – review & editing.

